# Liposomal and Ethosomal Gels: From Design to Application

**DOI:** 10.3390/gels9110887

**Published:** 2023-11-09

**Authors:** Maddalena Sguizzato, Rita Cortesi

**Affiliations:** Department of Chemical, Pharmaceutical and Agricultural Sciences (DoCPAS), University of Ferrara, 44100 Ferrara, Italy; crt@unife.it

The use of lipid-based nanosystems for topical administration represents an innovative “green” approach, being composed of materials, defined as GRAS (generally recognized as safe), characterized by low toxicity, biocompatibility, and biodegradability [1]. For instance, vesicular lipid-based delivery systems, such as liposomes [2], ethosomes [3], and niosomes [4], are good candidates for topical applications of active compounds for their dimensions, as well as their similarities, with the epidermal lipids becoming useful in the treatment of different pathologies, mostly in cutaneous diseases [5,6]. The main obstacle for the use of lipid-based nanosystems is their fluidity, which can hamper their in situ permanence. Hence, the possibility to incorporate them into polymeric gelled matrices can enhance the spreadability and adhesiveness onto the skin, mucosae, or other topical districts for biomedical, pharmaceutical, or cosmetic purposes [7,8,9,10,11]. 

This Special Issue of the Gels journal, entitled “Liposomal and Ethosomal Gels: From Design to Application”, includes twelve articles describing the production, characterization, and application of different nanocarriers included in gel systems, providing an interesting overview on gelled nanosystems from their qualitiy by design studies to their in vivo activity evaluation. Different administration routes have been considered, such as the cutaneous, buccal, ocular, and auricular routes, offering a wide spectrum of applications.

Notably, in the first research paper, the optimization of a PEGylated liposomal formulation by the quality by design (QbD) technique has been assessed to investigate the influence of lipid concentration on particle size, encapsulation efficiency, and in vitro release. The selected PEGylated Brucine liposomal emulgel based on jojoba oil showed an improved skin permeation, reflected in a significant anti-inflammatory effect (contribution 1).

An alternative lipid-based formulation, described by Khan et al. for the treatment of atopic dermatitis, revealed that the gel formulation embedding lipid nanoparticles by means of glycerol and sodium alginate maintained the same drug permeation level of the un-thickened nanoparticles, increasing the drug retention thanks to the gel’s bioadhesive properties (contribution 2). 

Vesicular nanosystems, namely, niosomes, containing ginger extract have been incorporated within the emulgel to obtain a transdermal delivery (contribution 3). The optimization of both niosomes and emulgel productions, obtained by applying QbD technique, demonstrated satisfactory physico-chemical characteristics and formulation enhancements of both skin permeability performance and anti-inflammatory effects due to the synergistic interaction between sesame oil and ginger extract from niosomes–sesame oil-based emulgel. In addition, Shabery et al. (contribution 4) incorporated a niosomal formulation for skin application within the patented palm oil base Hamin-C^®^. In vitro drug permeability was assessed by a Strat-M™ membrane, revealing higher permeability of both lidocaine and prilocaine when formulated with a cold process. 

Niosomes have been also considered for the encapsulation of natural phenolic compounds by using alternative xanthan gum or the thermoreversible polymer Poloxamer 407 as gelling agents to favor cutaneous applications (contribution 5). The preformulative study investigated the influence of the non-ionic surfactant and the hydration medium on the morphology, encapsulation efficiency, and in vitro release. The results showed that both the hydration phase and the type of thickening agent were able to control the drug diffusion. Niosomal gels based on xanthan gum revealed higher retention on the application site and no irritative reactions during in vivo patch tests in 95% of cases.

Among vesicular nanosystems, ethosomes represent an intriguing tool for topical applications. They have been considered for the encapsulation of rosehip extract to improve the stability of bioactive compounds, and their inclusion into hyaluronic acid gel allows for obtaining a suitable formulation for cosmetic use (contribution 6). The produced extract-loaded ethosomes showed small sizes, low polydispersity, and good entrapment efficiency and great stability during the time. The permeation study was performed through the artificial biomimetic barrier Permapad^®^, confirming the increasing of extract permeability when delivered by ethosomes. Lastly, the in vitro release studies, conducted on ethosomes and ethosomal–gel formulations, showed that encapsulation delayed the release of the extract. 

In addition to the common use of gel formulations for skin applications, buccal administration has been considered as an innovative application site for local or systemic effects.

Another effective emulgel, optimized by Central Composite Design (CCD) with the QbD method, was described by Iyer and colleagues. (contribution 7). The characterization of clove/cinnamon extracts-loaded emulgels showed globule sizes around 321 nm, contents of each extract around 96%, and good viscosity, spreadability, and extrusion properties. Additionally, the total release of loaded drugs demonstrated efficient anti-fungal potential in counteracting Candida-associated denture stomatitis. Clinical trials confirmed the effectiveness of the treatment with better taste acceptability and no side effects. 

The above-mentioned advantages of gel formulations (e.g., adhesiveness, spreadability, and sustained release) can be considered also to achieve the increasing of drug bioavailability. A carboxymethyl cellulose/hydroxypropyl cellulose (CMC/HPC) composite mixture was selected to produce an innovative formula for buccal applications (contribution 8). Carvedilol-loaded bilosomes, with spherical shapes and being 217 nm in diameter, showed a sustained drug release and high buccal permeability across sheep buccal mucosa. Their incorporation into a CMC/HPC nanosponge allowed for increasing the mucoadhesion of the formulation and to control the drug release thanks to the swelling ratio. The overall result was the management of hypertension with superior cardio-protective effects.

An oral in situ gel for the sustained delivery of Buspirone hydrochloride (BH) was developed to achieve a reduced daily dose frequency for the treatment of pediatric anxiety (contribution 9). Mucoadhesive gel was produced, utilizing alginates exhibiting sol-to-gel phase transitions due to pH changes. The formulation, optimized by a QbD study, resulted in an increased bioavailability of the drug as compared to the solution.

Furthermore, Zafar et al. described the use of gelled–lipid nanoparticles for the treatment of bacterial conjunctivitis after ocular application (contribution 10). Nanostructured lipid carriers (NLC) containing erythromycin were loaded into an in situ gel composed of carbopol and chitosan combination. The nanometric size and the high entrapment efficiency allowed to obtain high release and permeation of drugs, evaluated both in vitro and ex vivo on goat corneas, ensuring better antimicrobial activities. The selection of gelling polymers showed an improvement in precorneal residence time and tolerability, in terms of hydration, irritation, and isotonicity. 

Likewise, the treatment of ocular diseases was achieved with the development of a sol–gel system composed of Azithromycin-loaded lipid nanocarriers embedded in a thermosensitive gelling agent (contribution 11). The suitability of the formulation as ocular delivery system was assessed in vitro and ex vivo, demonstrating good corneal permeation, ocular tolerance as isotonic and non-irritant, and increased antimicrobial activity.

Lastly, the use of gel formulations has been investigated also for auricular delivery. A biopolymer lipid hybrid microcarrier was investigated for enhanced local Dexamethasone delivery and sustained release at the round window membrane level of the middle ear for the treatment of sensorineural hearing loss (SNHL) (contribution 12). In particular, polysaccharide–protein and pectin–bovine serum albumin were combined with lipids, such as Lipoid S100 and dimethyl-dioctadecyl-ammonium bromide, to obtain a hybrid biopolymer–liposome system. The presence of pectin hydrogel in the shell of the microparticles allowed to increase the microparticles’ stability profiles and their swelling behavior in aqueous environments. The sustained release provided by the formulation has been assessed by in vitro release studies that represent a fundamental condition for the prospective in vivo experiments. 

In conclusion, the application of gel formulations can reach different districts, offering controlled therapeutic effects. The optimization of the described drug delivery systems as a result of the QbD study led to shedding light on their advantages and drawbacks. 

Hence, the importance of gel formulations to increase residence time and adhesiveness of the investigated nanosized delivery systems has been largely demonstrated.
